# Vγ9Vδ2 T-Cell Polyfunctionality Is Differently Modulated in HAART-Treated HIV Patients according to CD4 T-Cell Count

**DOI:** 10.1371/journal.pone.0132291

**Published:** 2015-07-10

**Authors:** Rita Casetti, Gabriele De Simone, Alessandra Sacchi, Alessandra Rinaldi, Domenico Viola, Chiara Agrati, Veronica Bordoni, Eleonora Cimini, Nicola Tumino, Francesca Besi, Federico Martini

**Affiliations:** Laboratory of Cellular Immunology, “Lazzaro Spallanzani” National Institute for Infectious Diseases IRCCS, Rome, Italy; University of Cape Town, SOUTH AFRICA

## Abstract

Alteration of γδ T-cell distribution and function in peripheral blood is among the earliest defects during HIV-infection. We asked whether the polyfunctional response could also be affected, and how this impairment could be associated to CD4 T-cell count. To this aim, we performed a cross-sectional study on HIV-infected individuals. In order to evaluate the polyfunctional-Vγ9Vδ2 T-cell response after phosphoantigen-stimulation, we assessed the cytokine/chemokine production and cytotoxicity by flow-cytometry in HAART-treated-HIV+ persons and healthy-donors. During HIV-infection Vγ9Vδ2-polyfunctional response quality is affected, since several Vγ9Vδ2 T-cell subsets resulted significantly lower in HIV+ patients in respect to healthy donors. Interestingly, we found a weak positive correlation between Vγ9Vδ2 T-cell-response and CD4 T-cell counts. By dividing the HIV+ patients according to CD4 T-cell count, we found that Low-CD4 patients expressed a lower number of two Vγ9Vδ2 T-cell subsets expressing MIP-1β in different combinations with other molecules (CD107a/IFNγ) in respect to High-CD4 individuals. Our results show that the Vγ9Vδ2 T-cell-response quality in Low-CD4 patients is specifically affected, suggesting a direct link between innate Vγ9Vδ2 T-cells and CD4 T-cell count. These findings suggest that Vγ9Vδ2 T-cell quality may be indirectly influenced by HAART therapy and could be included in a new therapeutical strategy which would perform an important role in fighting HIV infection.

## Introduction

Human γδ T-cells are critical components of the innate immune system and play important roles in the early response to invading pathogens. Vγ9Vδ2 T-lymphocytes, the major population among γδ T-cells, display beneficial roles, as they are able to control HIV infection [1] and exhibit antiviral potential through their cell-lytic function and cytokine/chemokine secretions [2]. Alteration of γδ T-cell distribution in the peripheral blood of HIV-infected persons is the earliest defect in cellular immunity after infection [3]. Although the quantity and the quality of γδ T-cells have been found to generally decrease with the advancement of HIV infection [4], the antiviral functions of Vγ9Vδ2 T-cells can be enhanced by stimulation with phosphoantigen [5], suggesting a novel target for therapeutic strategies.

Recently, it was demonstrated that polyfunctional αβ T-cells are strongly associated with natural control of HIV disease, being higher in HIV-infected persons who spontaneously control the virus without any treatment regimen (eg. Long-Term Non-Progressor and Elite Controller) [6]. However, the ability of HAART treatment to restore polyfunctionality in T-cells is still controversial [7,8].

In the present paper, for the first time, Vγ9Vδ2 T-cell polyfunctionality has been evaluated by a cross-sectional study analyzing HIV-infected individuals under on HAART therapy, with different CD4 cell and viral load outcomes.

Our study demonstrated for the first time that, similar to αβ T-cells, HIV infection heavily modulates Vγ9Vδ2 T-cell polyfunctional response, and this effect was associated to CD4 T-cell count. Moreover, we identified two Vγ9Vδ2 T-cell subsets that could be considered as negative prognostic factors to disease progression. A longitudinal study is in progress to confirm this hypothesis.

## Materials and Methods

### Study participants

Fifty two HIV+ patients under HAART therapy from the “Lazzaro Spallanzani” Institute for Infectious Diseases in Rome were enrolled in the study. Healthy HIV-uninfected age and gender-matched individuals were included as controls. Table 1 shows the clinical data of the patients at the time of enrolment, grouped according to CD4 T-cell count in High-CD4 (H-CD4, >200 CD4 cells/mm^3^) and Low-CD4 (L-CD4, <200 CD4 cells/mm^3^) patients. The mean ± s.d. of CD4 counts was 901.8 ± 335.0 cells/mm^3^ for H-CD4 patients and 82.2 ± 44.8 cells/mm^3^ for L-CD4 patients. Considering the total number of patients, the mean ± s.d. of plasma HIV RNA was 132,116 ± 504,600 copies/mL.

**Table 1:** Clinical Characteristic of Patients at the Time of Blood Sampling.

Patients	Sex	Age (years) (mean±sd)	CD4 count (cells/mm^3^) (mean±sd)	Viral load (copies/mL)	Time on HAART (years)
17H-CD4^a^	11 Male 6 Female	40.8**±**10.1	901.8±335.0	11 ND^b^ 2 <40 58287.75**±**39905.1	16≤10 1>10
35L-CD4^c^	23 Male 12 Female	43.6±10.1	82.2±44.8	8 ND4 <40 331834.3±783806.1	34≤10 1>10

^a^patients with >200 CD4 cells/mm^3^;

^b^ND: Not Detected;

^c^patients with <200 CD4 cells/mm^3^.

Anonymised residual samples for routine CD4 analysis were used for the study. The study was approved by the “Lazzaro Spallanzani” National Institute for Infectious Diseases Ethical Committee (protocol 78 dated Nov.21, 2013), and a signed informed consent was obtained from the enrolled persons.

#### Whole blood stimulation and Vγ9Vδ2 T-cell flow cytometry analysis

**Fig 1:**
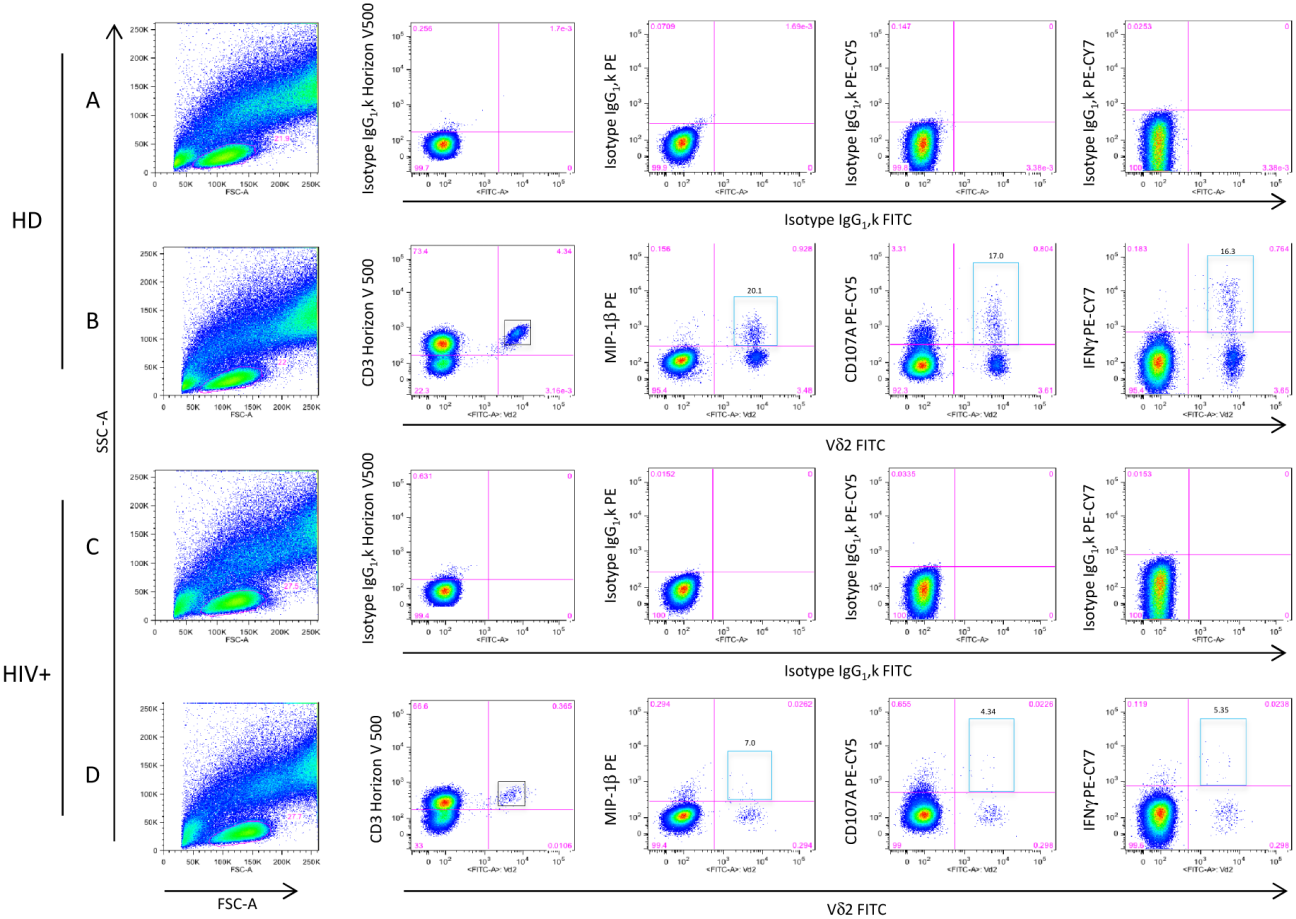
Multi-parameter flow cytometric analysis of the functional profile of Vγ9Vδ2 T-cells. Representative dot plots of functional parameters (MIP-1β, CD107A, IFNγ panels B and D) and the respective Isotype control of antibodies (panels A and C) were shown in healthy donors (HD) and HIV-infected patients (HIV+) after stimulation with Picostim. For the gating strategy, after the lymphocyte population was gated sequentially, CD3+ and Vγ9Vδ2 T-cell events were defined, and gates for each respective function were made (panels B and D, light blue gates) using combinations that provided optimal separation. Single function gates were set based on the negative control (unstimulated) samples and were placed consistently across samples. We used the Boolean gate platform to create the full array of possible combinations, equating to 8 (2^3^) response patterns when testing three functions. Data are reported after background correction. Numbers on the light blue gates indicate the percentage of functional Vγ9Vδ2 T-cells among the total Vγ9Vδ2 T-cells (black boxes).

Whole blood diluted with complete medium (1:2) was stimulated with Picostim, a specific Vγ9Vδ2 T-cell antigen (80nM, kindly gifted by Dr. Helene Sicard, Innate Pharma SA, Marseille, France). In all conditions CD107a-PE-Cy5 antibody (20 μl/mL of diluted whole blood, according to the manufacturer’s instructions, Becton-Dickinson BD, Buccinasco, Milan, Italy) and Brefeldin A (20 μg/mL, Sigma-Aldrich, Gallarate, Milan, Italy) were added at the beginning of culture and after 1 hour of stimulation, respectively. After 18 hours, cells were labeled with anti-CD3 Horizon V500 and anti-Vδ2 FITC (10 min. at 4°C); then a lysing/fixing solution was added to the cells, according to the manufacturer’s instructions (BD). After washing (PBS/BSA/NaN3 buffer), cells were incubated for 20 min. at room temperature (RT) with antibodies for intracellular staining (MIP-1β-PE and IFNγ PE-Cy7) prepared in a permeabilizing buffer, and then washed with 0.1% saponin/PBS/BSA/NaN3 solution. All antibodies were purchased from Becton-Dickinson (BD, Buccinasco, Milan, Italy). Samples were acquired on FACSCanto II flow cytometer (BD) and analyzed using FlowJo 6.4.7 software (TreeStar, Olten, Switzerland). FlowJo software, through the Boolean gate platform, allowed for creating the full array of possible combinations, equating to 7 (2^3^-1) response patterns for three functions, excluding the fully negative subset (Fig 1). After background correction, SPICE Software was used to graph and analyze the data [9].

### Statistical analysis

The Mann–Whitney U test and Spearman R test were used to compare group medians and determine a correlation, respectively (Prism version 5, GraphPad Software, La Jolla, California, USA). The statistical analysis of the polyfunctional responses was performed in SPICE version 5.22 using a permutation test based on χ2 test to compare pie charts. A p value less than 0.05 was considered statistically significant.

## Results

### CD107a- IFNγ- or MIP-1β-expressing Vγ9Vδ2 T-cell subsets decreased during chronic HIV infection

**Fig 2:**
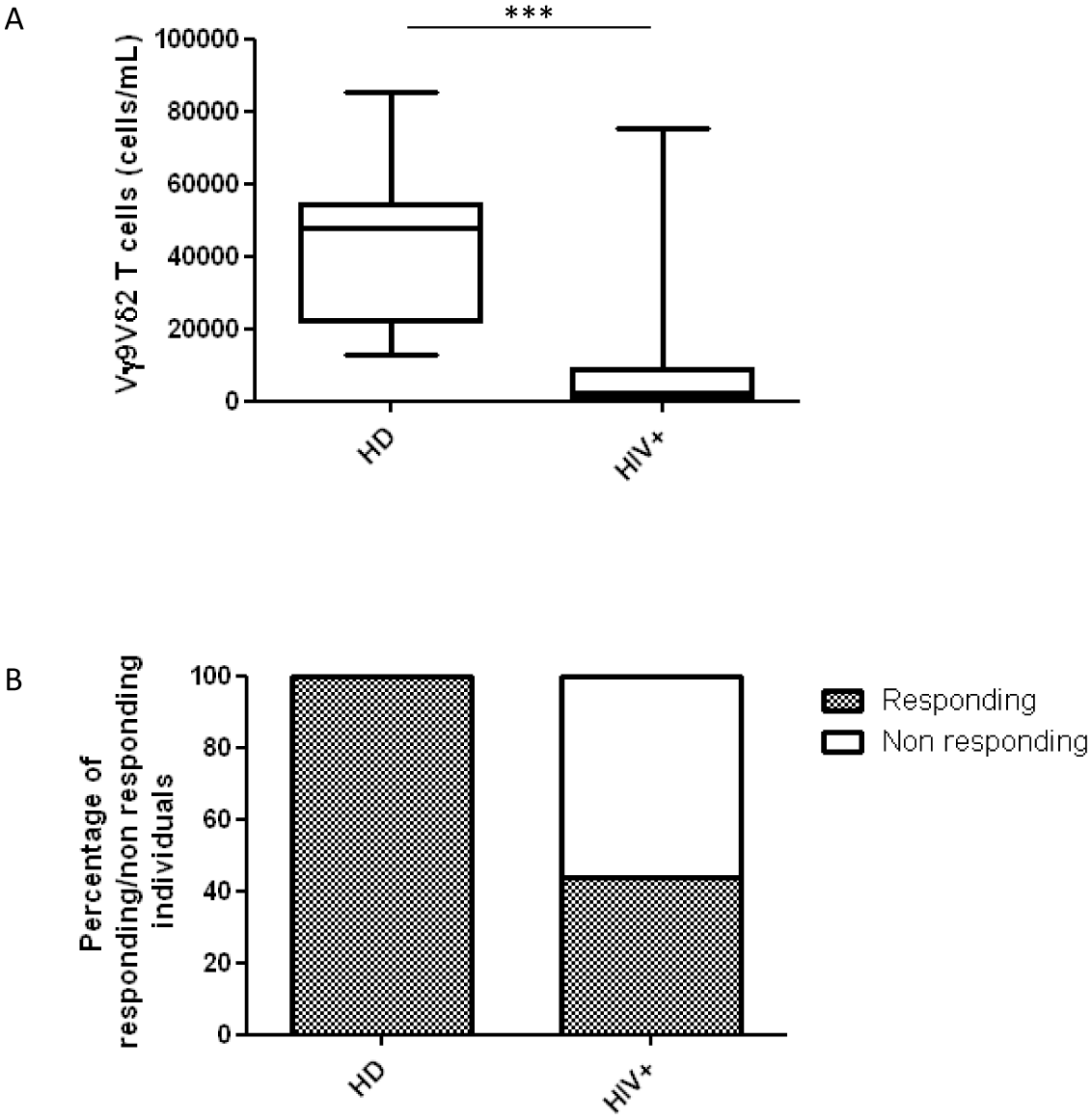
Responding Vγ9Vδ2-individuals decreases among HIV-infected patients who present lower Vγ9Vδ2 T-cell number than HD. Flow cytometer analyses were made to show (A) the absolute number of Vγ9Vδ2 T-cells from *ex vivo* HIV-infected patients (HIV+) and healthy donors (HD). The results are shown as median and interquartile range (box plot), and vertical lines show the minimum and maximum values; (B) the percentage of individuals among the HIV+ patients and HD having responding Vγ9Vδ2 T-cells after Picostim stimulation. Responding individuals, grey bar; Non-responding individuals, white bar. The Mann–Whitney U test was used to compare group medians. A p value less than 0.05 was considered statistically significant, ***p<0.001.

**Fig 3:**
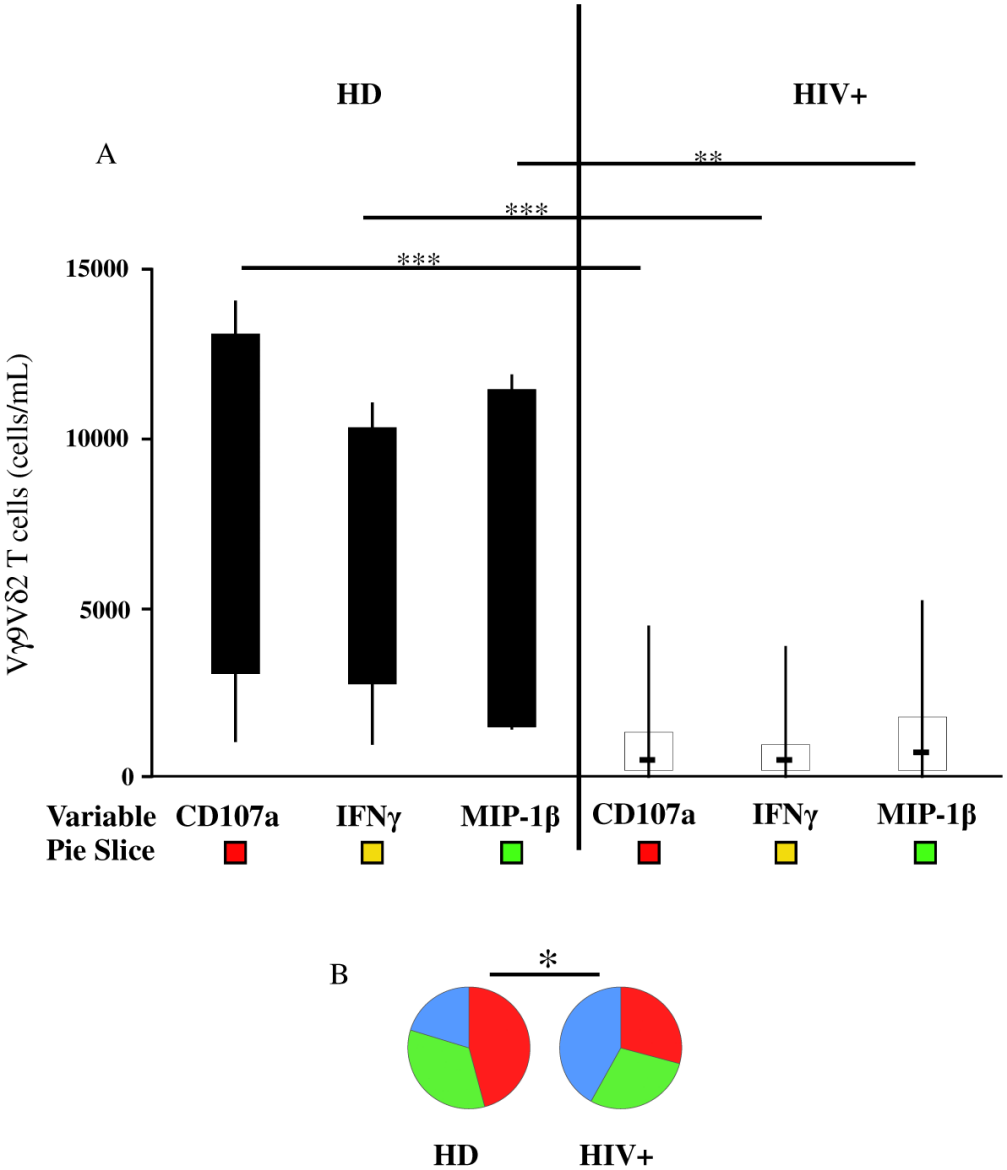
CD107a-, IFNγ- and MIP 1β-producing Vγ9Vδ2 T-cell subsets decrease in HIV+ patients. According to the type of function, Vγ9Vδ2 T-cell response was analyzed in the healthy donors and compared to the HIV+ patients by flow cytometry after Picostim stimulation. (A) The absolute number of different Vγ9Vδ2 T-cell subsets according to the type of functions expressed (CD107a, IFNγ and MIP-1β). The results are shown as median and inter-quartile range (box plot), and vertical lines show the minimum and maximum values. (B) Summary pie chart; each sector of the pie chart is matched to the colored squares shown below the bar graphand shows the median percentages of responding Vγ9Vδ2 T-cells grouped according to the type of function analyzed. The Mann–Whitney U test was used to compare group medians and a permutation test based on χ^2^ test to compare pie charts. A p value less than 0.05 was considered statistically significant, ***p<0.001; **p<0,01; *p<0.05; HD = healthy donors, black bar; HIV+ = HIV-infected individuals, white bar.

First we analyzed the absolute number of Vγ9Vδ2 T-cells in the HD and HIV+ patients. As known, Vγ9Vδ2 T-cells were significantly lower in the HIV+ patients than HD (p<0.0002, Fig 2A). Then, PBMC from the HD and HIV+ subjects were stimulated with Picostim, and the ability of Vγ9Vδ2 T-cells to produce cytokines and chemokines and to exert cytotoxicity were analyzed by flow cytometry. A total response, measured as the sum of all unique functional Vγ9Vδ2 T-cell subsets performing one or more functions, was observed in 100% of the HD and 44.2% of the HIV+ patients (Fig 2B). Thus, we analyzed the Vγ9Vδ2 T-cell polyfunctional subsets only in responding individuals. Specifically, in HD and responding HIV+ patients, we analyzed the contribution to the total response of Vγ9Vδ2 T-cells according to the cytokines or chemokines produced, and the cytotoxic molecules expressed (CD107a). In HD, a consistent number of stimulated Vγ9Vδ2 T-cells expressed CD107a, and produced IFNγ and MIP-1β (Fig 3A). Notably, in the HIV+ patients, a significant reduction of CD107a-, IFNγ- or MIP-1β-producing Vγ9Vδ2 T-cell subsets was observed (p = 0.0006, p = 0.0006, and p = 0.0052, respectively; (Fig 3A). Accordingly, a significant difference between the HD and HIV+ patients was also confirmed by analyzing the global cytokine profile (p = 0.0127; Fig 3B).

### Three-, bi- and mono-functional Vγ9Vδ2 T-cell subsets decreased in HIV-infected patients

**Fig 4:**
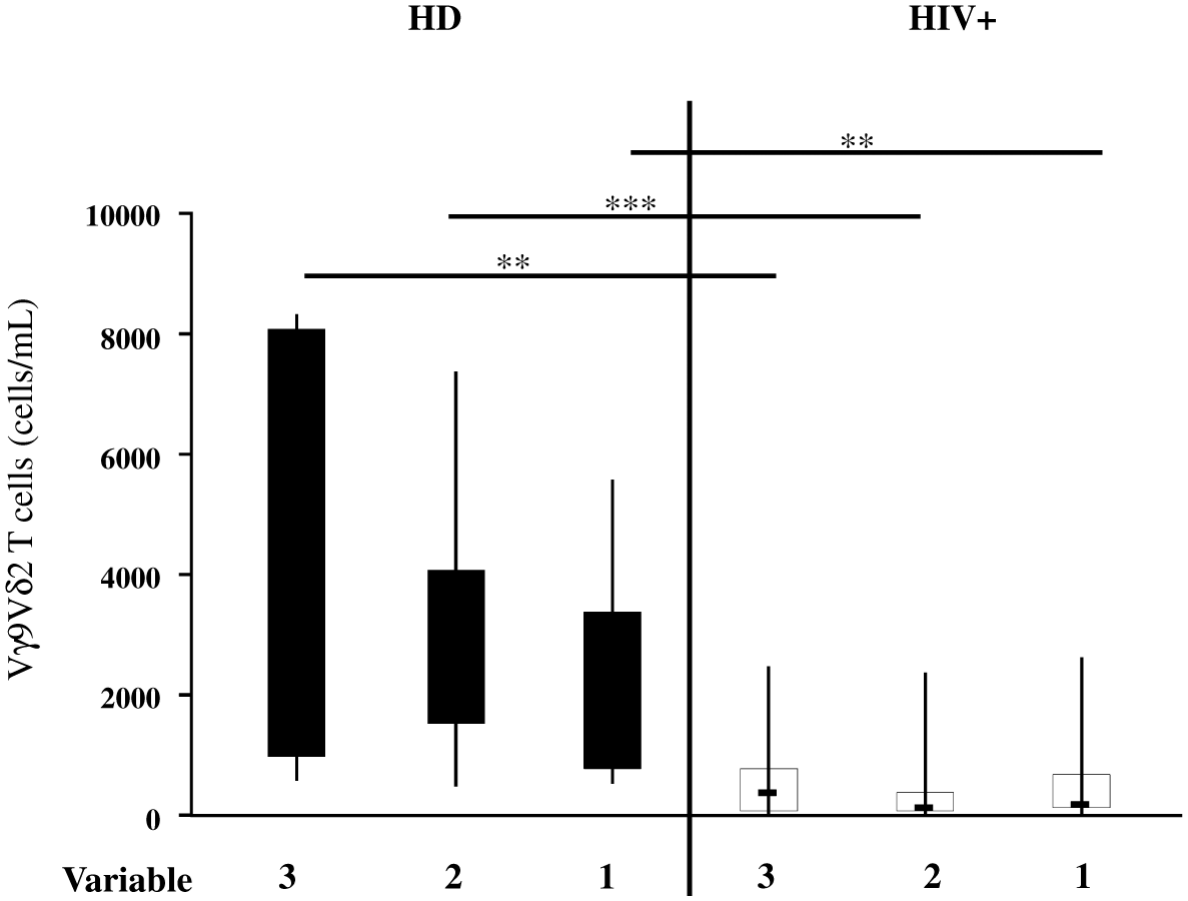
Three-, bi- and mono-functional Vγ9Vδ2 T-cell subsets decrease in HIV+ patients. According to the number of functions, Vγ9Vδ2 T-cell response was analyzed in the healthy donors and compared to the HIV+ patients by flow cytometry after Picostim stimulation. The graph shows the absolute number of different Vγ9Vδ2 T-cell subsets according to the number of simultaneous functions expressed. The results are shown as median and inter-quartile range (box plot), and vertical lines show the minimum and maximum values. The Mann–Whitney U test was used to compare group median. A p value less than 0.05 was considered statistically significant, ***p<0.001; **p<0,01; *p<0.05; HD = healthy donors, black bar; HIV+ = HIV-infected individuals, white bar.

Our data showed that, similar to αβ T-cells, Vγ9Vδ2T-cells were able to produce more than one function. We therefore analyzed the contribution to the total response of Vγ9Vδ2 T-cells according to the number of functions (1, 2 or 3 functions) in the HD and HIV+ patients. The polyfunctional profile analysis showed a similar number of three-, bi- and mono-functional Vγ9Vδ2 T-cell subsets in the HD as well as in the HIV+ patients (Fig 4). Interestingly, in the HIV+ patients, a significant decrease of three-, bi-, and mono-functional Vγ9Vδ2 T-cell subsets was observed in respect to the HD (p = 0.0024, p = 0.0005 and p = 0.0020, respectively, (Fig 4)).

### Contribution of each unique functional subset to the global response

**Fig 5:**
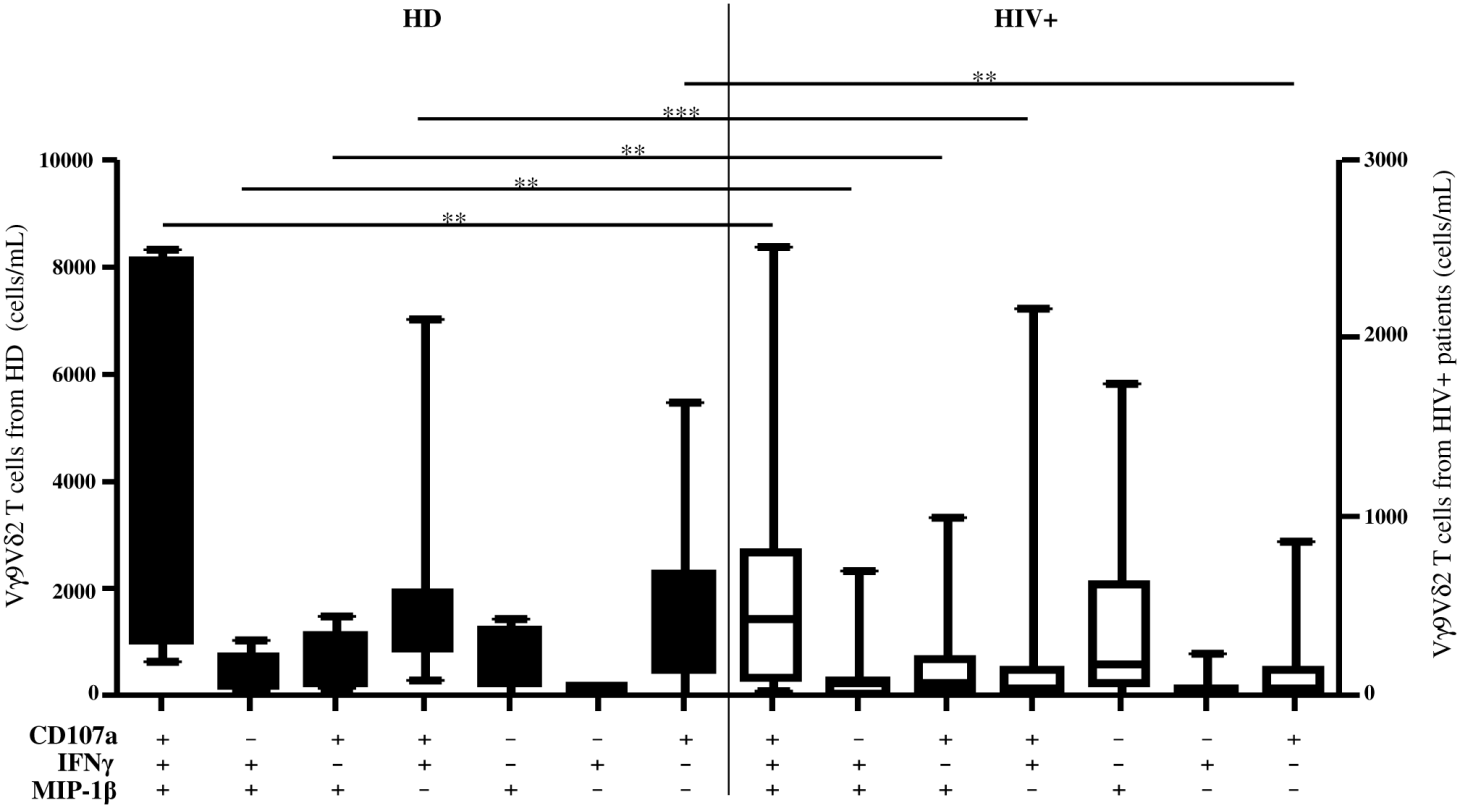
Modulation of unique Vγ9Vδ2 T-cell subsets during HIV infection. Seven unique Vγ9Vδ2-cell subsets derived from all possible combinations of the three parameters were analyzed from the whole blood of healthy donors and compared to the HIV+ patients by flow cytometry after Picostim stimulation. The graph shows the absolute number of different unique Vγ9Vδ2T-cell subsets according to the particular combination of functions expressed. The results are shown as median and inter quartile range (box plot), and vertical lines show the minimum and maximum values. The Mann–Whitney U test was used to compare group medians. A p value less than 0.05 was considered statistically significant, ***p<0.001; **p<0,01; *p<0.05; HD = healthy donors, black bar; HIV+ = HIV-infected individuals, white bar.

Finally, we analyzed the contribution of each unique functional subset to the global response according to the particular combination of expressed molecules. We obtained seven functional subsets derived from the combination of three molecules. As shown in Fig 5, after Picostim stimulation, in the HIV+ patients, a significant decrease was observed in all functional subsets with the exception of one subset (MIP-1β+) that was comparable to the HD (Fig 5). The IFNγ subset was not considered in the analysis due to its very low frequency.

### Both total and functional Vγ9Vδ2 T-cells correlate with CD4 T-cell count

**Fig 6:**
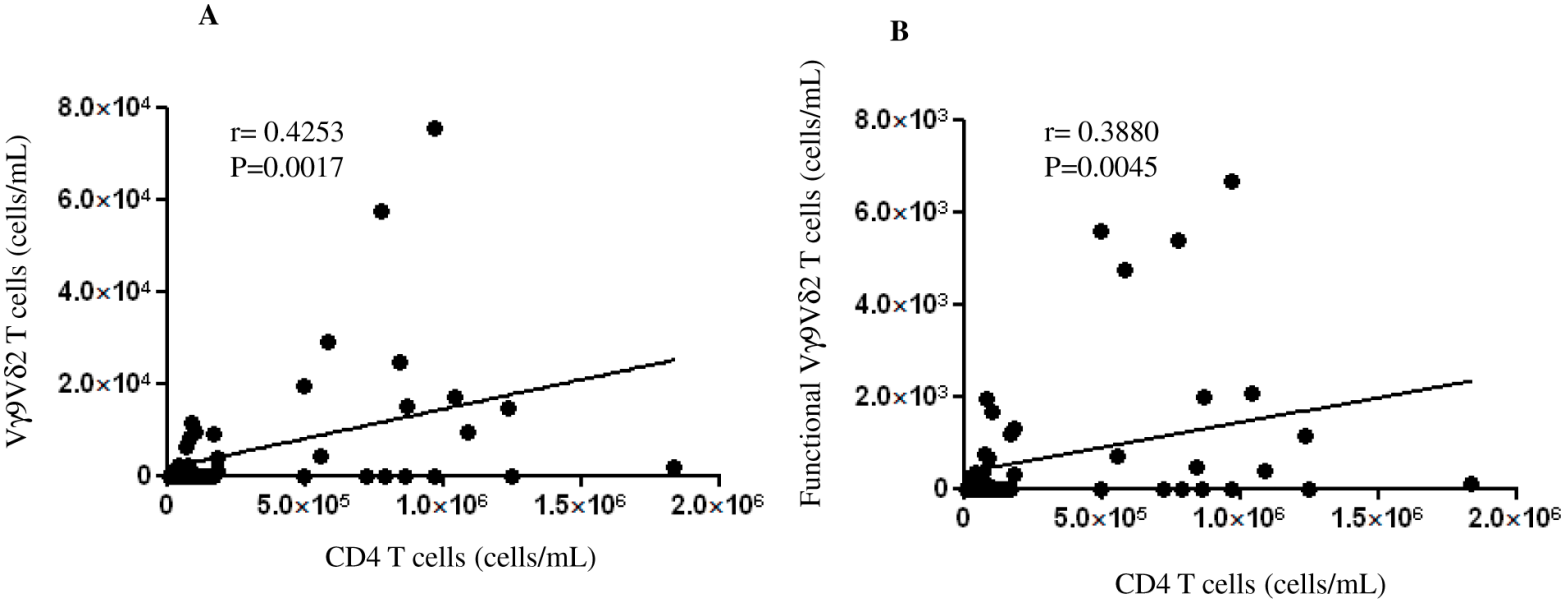
Total and functional Vγ9Vδ2 T-cells correlate with CD4 cell count. Total (A) and functional (B) Vγ9Vδ2 T-cells from the HIV+ patients were correlated with CD4 cell count. Correlation statistics were analyzed using the Spearman R test and a p value less than 0.05 was considered statistically significant, ***p<0.001; **p<0,01; *p<0.05.

We wondered whether the Vγ9Vδ2 T-cell number and functional capability was associated to CD4 T-cell count and/or plasma viral load. We found that CD4 T-cell count was positively correlated with both the Vγ9Vδ2 T-cell absolute number (p = 0.0017, Fig 6A) and Vγ9Vδ2 T-cell functional capability (p = 0.0045, Fig 6B). In contrast, no correlation with viral load was observed (data not shown).

### Patients with low CD4 cell counts showed a lower number of two Vγ9Vδ2 T-cell subsets expressing MIP-1β in different combinations with CD107a and IFNγ

**Fig 7:**
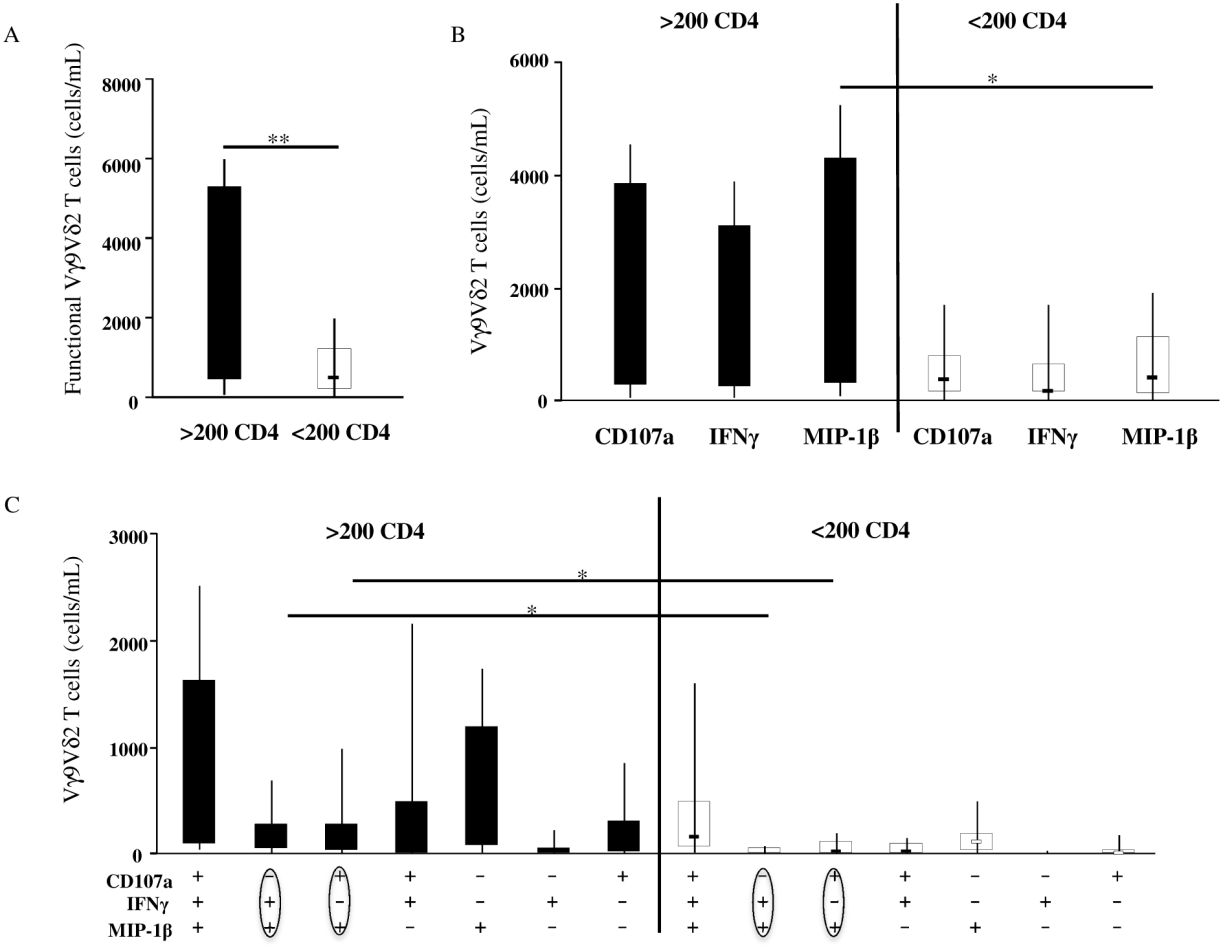
Low-CD4-Patients showed a lower number of two Vγ9Vδ2 T-cell subsets expressing MIP-1β. Patients were divided into two groups according to CD4 cell count and Vγ9Vδ2 T-cell function was analyzed by flow cytometry after Picostim stimulation. (A) Vγ9Vδ2 T-cell total response. (B) The absolute number of different Vγ9Vδ2 T-cell subsets according to the to the type of functions expressed (CD107a, IFNγ and MIP-1β). (C) The absolute number of seven different unique Vγ9Vδ2 T-cell subsets according to the particular combination of functions expressed.

Since a positive correlation between functional Vγ9Vδ2 T-cells and CD4 cells was observed, we further analyzed this issue by sub-grouping the HIV+ patients into High-CD4 (H-CD4) and Low-CD4 (L-CD4) according to CD4 cell counts. As shown in Fig 7A, the total response was significantly lower in the L-CD4 patients when compared to the H-CD4 patients (p = 0.0078), confirming a strict correlation between the quality of immune reconstitution and the functional quality of Vγ9Vδ2 T-cells.

Finally, we analyzed the contribution to the total response of Vγ9Vδ2 T-cells according to the cytokines produced, as well as the contribution of each unique functional subset in the High- and Low-CD4 patients. We found that the total MIP-1β expression was lower in the L-CD4 patients when compared to the H-CD4 subjects (p = 0.0455, Fig 7B). Interestingly, we found a significant statistical difference in the CD107a+MIP-1β+ and IFNγ+MIP-1β+ subsets (p = 0.0287 and p = 0.0137, respectively. Fig 7C), suggesting their possible use as treatment outcome markers.

The results are shown as median and inter-quartile range (box plot), and vertical lines show the minimum and maximum values. The Mann–Whitney U test was used to compare group medians and a p value less than 0.05 was considered statistically significant, ***p<0.001; **p<0,01; *p<0.05; >200 CD4 = patients with >200 CD4 cells/mm^3^; <200 CD4 = patients with <200 CD4 cells/mm^3^.

## Discussion

Vγ9Vδ2 T-cells are a critical component of innate immunity. After phosphoantigen stimulation they are able to secrete cytokines and chemokines and to exhibit cytotoxicity activity against HIV-infected cells, thus exhibiting beneficial roles in controlling HIV infection [1].

HIV infection causes an alteration of γδ T-cell distribution leading to a specific depletion of Vγ9Vδ2 T-cells in peripheral blood [3,10]. Although with contrasting results, long-term treatment with HAART led to only partially recovery this population [11,12]. However, little is known about the Vγ9Vδ2 T-cell polyfunctional response capability, and about its role during antiretroviral treatment.

It was largely demonstrated that αβ T-cell polyfunctionality is correlated to HIV disease control, being higher in HAART-naïve patients who spontaneously control disease progression [8] and in HAART-treated patients who reduce the viral load to undetectable levels [13,14]. Our results showed that HIV+ patients exhibit lower CD107a-, IFNγ- and MIP-1β producing Vγ9Vδ2 T-cell subsets than healthy donors. Many authors demonstrated a general decrease of γδ T-cell functions during HIV infection [5,15]. Secretion of perforin and granzyme B is one of the pathways through which γδ T-cells exert direct anti-infection response [16]. We demonstrated that Vγ9Vδ2 T-cells in HIV+ patients have fewer cells secreting perforin and granzyme, detected by measuring CD107a expression [17]. The release of cytokines and chemokines is another important immunoregulatory function of γδ T-cells. Vγ9Vδ2 T-cells respond to phosphoantigen stimulation by producing type I cytokines including IFNγ. This cytokine, together with TNFα, polarizes the immune response toward type I immunity, thus possibly increasing the capacity to control the virus. Moreover, IFNγ producing γδ T-cells could be important in immune homeostasis, since IFNγ has been found to antagonize Treg functions [18]. Our data are in agreement with other authors [19,20] showing a γδ T-cells-impaired functional response from HIV+ patients that is only partially restored with HAART treatment. Moreover, Vγ9Vδ2 T-cell activation with phosphoantigens causes rapid induction of chemokines such as MIP-1β which are able to suppress HIV replication *in vitro* [21]. As previously demonstrated [22] Vγ9Vδ2 T-cells during HIV infection were found to be anergic and unable to perform their effector function, with a reduced production of cytokines including IFNγ. Moreover, Garcia et al. [23] demonstrated a loss of Th1 responses by γδ T-cells during the course of HIV infection, contributing to clinical immunodeficiency.

Here, for the first time, we demonstrated both the capacity of Vγ9Vδ2 T-cells to simultaneously perform different functions, including cytokine/chemokine secretion and cytotoxicity activity in healthy donors, and their impairment during HIV infection. Specifically, we showed that three-, bi- and mono-functional Vγ9Vδ2 T-cell subsets decrease in HIV infected patients in respect to healthy donors, indicating that polyfunctional capability is affected during HIV infection. Limited data are available in literature regarding γδ T-cell polyfunctional capability. Bridgett Ryan-Payseur [24] demonstrated, in non-human primates, multi-effector-functional Vγ9Vδ2 T-cell responses against L. monocytogenes producing or coproducing Th1 and Th2 or Th17 cytokines, and direct lysis of L. monocytogenes-infected target cells. Restrepo et al. [25] showed a higher expression of bi-functional γδ T-cell subsets coproducing IFNγ and MIP-1β in HIV-exposed seronegative individuals than in HIV-infected partners and healthy donors. However, our data could not be compared with these data, because these authors stimulated γδ T cells with HIV peptides and not with constitutive phosphoantigens. Our data showed an alteration of some unique functional Vγ9Vδ2 T-cell subsets in HIV+ patients when compared to healthy donors, indicating that HIV infection may affect the specific quality of Vγ9Vδ2 T-cell subset response.

Our results are in line with Li et al. [4], by showing that both the number and functional quality of Vγ9Vδ2 T-cells correlate to CD4 cell counts. Specifically, we found that patients with low CD4 cell counts present a lower total response. Moreover, we found that these patients showed a decrease in MIP-1β levels.

Interestingly, we found that two specific unique functional subsets, both expressing MIP-1β (CD107a+MIP-1β+ and IFNγ+MIP-1β+), were modulated by CD4 cell counts which were inferior in patients with lower CD4 cell counts. It is known that MIP-1β production dominates HIV-specific CD8 T cell response [6], suggesting that this chemokine may be the best single indicator of HIV-specific CD8 T-cell frequency. We could propose that these specific subsets might be considered as possible prognostic factors of treatment failure.

In conclusion, our data support the notion that a specific modulation of Vγ9Vδ2T-cell response capability is present in HIV-infected individuals, and is correlated to the course of HIV disease. These results highlight a supplemental beneficial role of antiretroviral treatment in allowing also the recovery of innate Vγ9Vδ2 T-cells function.

As to the limitations of the present study, the limited number of HIV+-responding individuals suggests additional studies with larger cohorts, in order to confirm these results. Moreover, further studies including a longitudinal assessment of responses will help to elucidate whether Vγ9Vδ2 T-cell response capability modulations effectively contribute to protection in modifying HIV disease course, or whether they simply reflect a specific immune imbalance following HIV infection.
